# Proteomic profiling of the tumor microenvironment: recent insights and the search for biomarkers

**DOI:** 10.1186/gm529

**Published:** 2014-02-27

**Authors:** Sam Hanash, Mark Schliekelman

**Affiliations:** 1 Anderson Cancer Center, Bertner Avenue, Houston, TX 77030, USA; 2 Fred Hutchinson Cancer Center, Seattle, WA 98109, USA

## Abstract

Although gain of oncogene functions and loss of tumor suppressor functions are driving forces in tumor development, the tumor microenvironment, comprising the extracellular matrix, surrounding stroma, signaling molecules and infiltrating immune and other cell populations, is now also recognized as crucial to tumor development and metastasis. Many interactions at the tumor cell-environment interface occur at the protein level. Proteomic approaches are contributing to the definition of the protein constituents of the microenvironment and their sources, modifications, interactions and turnover, as well as providing information on how these features relate to tumor development and progression. Recently, proteomic studies have revealed how cancer cells modulate the microenvironment through their secreted proteins and how they can alter their protein constituents to adapt to the microenvironment. Moreover, the release of proteins from the microenvironment into the circulatory system has relevance for the development of blood-based cancer diagnostics. Here, we review how proteomic approaches are being applied to studies of the tumor microenvironment to decipher tumor-stroma interactions and to elucidate the role of host cells in the tumor microenvironment.

## The tumor microenvironment

**Figure 1:**
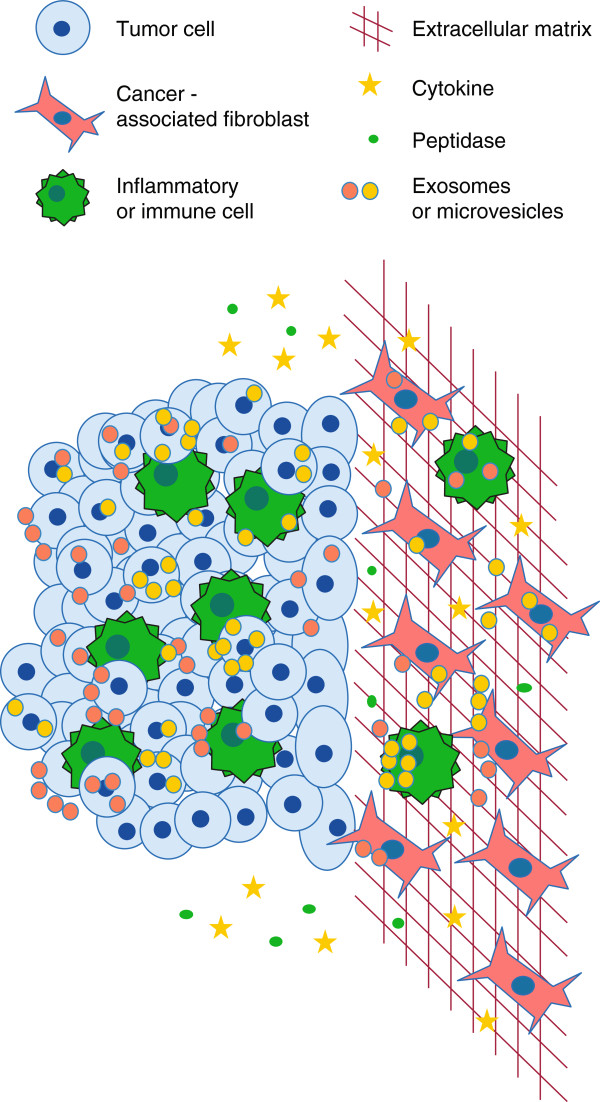
**Constituents of the tumor microenvironment.** A mix of tumor cells, cancer-associated fibroblasts and inflammatory or immune cells permeate the microenvironment. These cells produce the extracellular matrix and an assortment of soluble factors. Cross-talk between the resident cell populations is further contributed to by the release and/or uptake of exosomes and microvesicles loaded with cargo that includes nucleic acids, proteins and metabolites.

The tumor microenvironment is functionally important for tumor development and progression. It comprises multiple components: the extracellular matrix (ECM), surrounding stromal cells and infiltrating cells, and signaling molecules (Figure 1). Studies of the tumor microenvironment have involved model systems, both *in vitro* and *in vivo*, and the use of patient samples. The stroma, ECM and infiltrating cells interact with tumor cells, and have the capacity to both aid and hinder tumor development. The ECM is a structural network composed primarily of collagens, laminins and various glycoproteins. It serves a scaffolding function for tissue organization, in part through its physical properties but also by interacting with cells through cellular receptors and embedded signaling molecules [1,2]. Increased matrix stiffness enhances tumor-cell migration in many tumor types [3-5]. Interactions between integrins on the cell surface and matrix proteins provide signaling information that affects tumor cell proliferation, motility and other functions [6]. In addition to the structural ECM components, matricellular proteins that are largely produced by cancer-associated fibroblasts (CAFs) have dual roles as structural and signaling factors [7,8]. The stroma can be composed of a wide variety of cell types, including resident epithelial cells, fibroblasts, endothelial cells, pericytes and cells of the immune system [9,10]. Fibroblasts are primarily responsible for ECM production and participate in the wound-healing process [11]. Endothelial cells support vascular development and are essential for angiogenesis, which is also required for tumor growth. The majority of solid tumors are infiltrated with inflammatory cells, suggesting a host immune response to the tumor. Nevertheless, immune cells frequently fail to respond adequately to the tumor and are subverted to aid in tumor development.

Overall, it has become clear from recent studies that the tumor microenvironment has dual capacities to induce either a beneficial or an adverse effect on tumorigenesis. Recently, therapies have been developed that target tumor cells for destruction by the host immune system [12]. In this review, we discuss proteomic approaches that are being applied to studies of the tumor microenvironment in order to decipher tumor-stroma interactions and to elucidate the role of host cells in the microenvironment. We also discuss potential biomarker applications of proteins that are derived from tumor-microenvironment interactions.

## The reach of proteomics

**Figure 2:**
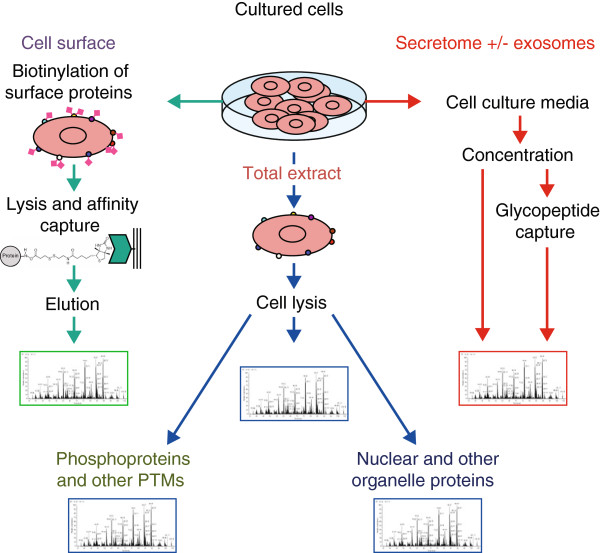
**Analysis of the proteome of cell populations by sub-compartment.** Proteomics is particularly informative when applied to individual cellular compartments, such as the cell surface, secretome, the nucleus or other organelles, for which isolation and analysis procedures are required. Moreover, aside from the identification of constituent proteins, there is a need to assess post-translational modifications (PTMs) of proteins, including major modifications such as phosphorylation and glycosylation, in addition to cleavage and proteolysis. Cell surface proteins (for example, receptors and antigens) may be captured through the use of lipid impermeable biotin followed by capture of surface proteins using monomeric avidin and subsequent mass spectrometry (MS) analysis. Proteins in cell culture media may be fractionated as intact proteins using chromatography followed by digestion of individual fractions and MS analysis. Alternatively, particulate material (for example, exosomes) may be first isolated from media, followed by their MS analysis separately from the soluble fraction in the media.

**Figure 3:**
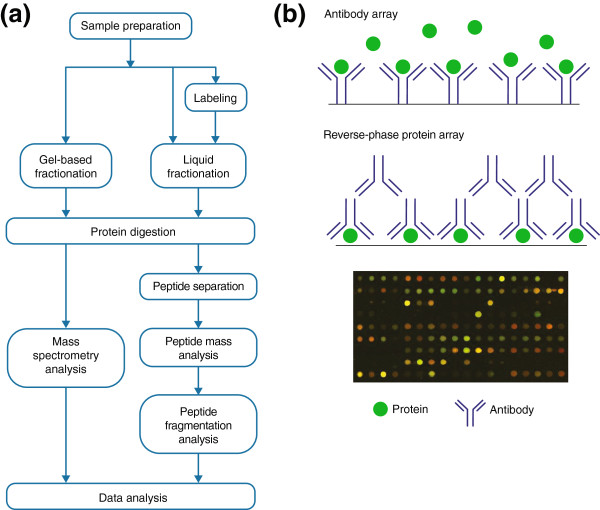
**Methodologies for proteomic analysis. (a)** Experimental workflow of common mass spectrometry (MS)-based proteomic approaches. Proteins from particular compartments or from whole-cell lysates may be separated using gel electrophoresis or chromatography. Individual fractions are subsequently digested, followed by MS of peptide mixtures. **(b)** Affinity-based proteomic analysis is generally applied to intact proteins. Antibodies with defined specificities may be arrayed on a glass slide or membrane followed by incubation with a lysate. Alternatively, with reverse phase protein arrays, lysates are spotted and incubated with individual antibodies that target a specific protein.

Advances in protein analysis by mass spectrometry (MS), coupled with front-end separations and peptide or protein labeling, have allowed comprehensive analysis of the protein complement of a cell, tissue or organ [13]. Proteins from whole-cell lysates, from particular cell-derived compartments such as exosomes [14], or from chromatographic or affinity-capture fractions (Figure 2) are subjected to enzymatic cleavage into peptides, followed by chromatographic separation and MS analysis (Figure 3a). Techniques for proteome analysis utilizing electrospray ionization (ESI) or matrix-assisted laser desorption ionization (MALDI) have advanced substantially [15]. With tandem MS analysis (MS/MS), an initial determination of the mass of a peptide is made (MS1) and a subsequent MS analysis (MS2) measures peptide fragments and provides accurate sequence information by matching to sequence databases. Biological samples have a high degree of complexity and protein concentrations vary across a wide dynamic range, complicating proteomic analysis [16]. To reduce complexity, samples can be fractionated or enriched in particular compartments prior to MS analysis [17]. MS may be combined with activity-based probes to determine not only the occurrence and quantification of proteins but also their functional activity [18].

Affinity-based proteomics involving capture agents, notably antibodies, can be utilized to facilitate high-throughput analysis of proteins or for confirmatory studies, for example, using tissue microarrays (Figure 3b) [19]. Antibodies may be spotted onto an array to allow capture and identification of their targets. Alternatively, with reverse-phase protein arrays, lysate samples are spotted onto arrays that are incubated with an antibody against a known target [20]. Proteins and peptides may also be spotted onto microarrays for their interrogation.

The depth of analysis currently achievable through proteomics is such that, given a sufficient number of cells, a protein product of virtually any expressed gene in a cell population should be identifiable. The challenge for proteomic studies of the tumor microenvironment stems from the cellular complexity of the microenvironment, necessitating separate analysis of individual cell types. Moreover, the insoluble nature of the matrix complicates analysis of intact proteins, a challenge that can be overcome by first digesting the matrix before MS of the resulting peptide mixture. MS tools are increasingly being utilized to characterize the numerous post-translational protein modifications that play a role in the tumor microenvironment - for example, through their impact on the cross-linking of matrix proteins [21]. Above all, for studies of the tumor microenvironment to be most informative, they must utilize the in-depth, quantitative proteomic methodologies that are currently available but largely restricted to specialized laboratories and centers.

## Insights from proteomic profiling of the tumor extracellular matrix

The ECM regulates intercellular communication and serves as a repository for cell signaling molecules, in addition to serving a structural scaffolding role for cells [22]. Proteomics has provided insights into constituents of the ECM and their regulation (Table 1). One approach involves stable isotope labeling of amino acids in cell culture (SILAC), which enables proteins that are newly produced by cells to be differentiated from culture media supplemented with serum [23]. Studies analyzing the proteins released from cell lines have defined ECM constituents that are produced by tumor cells. Combined liquid chromatography-tandem MS (LC-MS/MS) has been used to analyze proteins released into conditioned media, surface proteins and whole-cell lysates of murine lung cancer cells that have undergone epithelial-mesenchymal transition (EMT, a process that allows tumor cells with an epithelial phenotype to convert to mesenchymal cells). These analyses revealed increased expression of a number of ECM proteins that indicate that the tumor cells are shaping their microenvironment, including: fibronectin, which binds to integrin receptors; a member of the collagen family, collagen 6A1; and members of the laminin family of glycoproteins, laminins A5, B2, and C1 [24].

**Table 1:** Highlights from proteomic studies of the tumor microenvironment

	**Experimental system**	**Approach**	**Key proteins**
**Tumor ECM**	EMT in murine cancer cells [24]	MS of subcellular fractions	Fibronectin, collagens, laminins
	Metastatic melanoma cells [26]	Secretome MS	Fibronectin, ECM1, SPARC, osteopontin
	Hepatocarcinogenesis [29]	ECM MS	Collagen splice variants, nidogen, decorin, perlecan
	Cell attachment to collagen [34]	Phosphoprotein analysis	DBF4, GRK6, PAK2, FAK/ PTK2
	Breast cancer metastasis [34]	MS	Cystatin-M
	Tumor invasiveness [41]	Antibody libraries	hsp90α
**Tumor-stroma interactions**	Tumor versus normal stroma [45]	Laser capture + MS	CapG (actin-regulating protein)
	CAFs [49]	MS of cell lysates	Caveolin-1
	MMP7 effect of CAFs [50]	MS of cell lysates	IGFBP5
	Lung cancer TME [54]	Cytokine assays	CXCL1, IL-18
	Stromal cell secretome [60]	Antibody arrays	HGF
**Immune cells in the tumor microenvironment**	Mesothelioma effusions [67]	Antibody arrays	HGF, MIP-1d, MIP-3a, NAP-2
	Ovarian tumor macrophages [68]	MS of supernatants	14-3-3 zeta
	CD45RA + versus CD45RO + T cells [70]	MS of cell lysates	Cell redox proteins
	MDSCs + IL1B [72]	MS of cell lysates	FAS pathway and caspase network

Some studies have focused on the ECM, others on tumor-stroma interactions and on immune cells infiltrating the tumor microenvironment. These studies have utilized various experimental systems applied to different tumor types. Some of the key proteins that have been found to be involved are highlighted, illustrating the numerous protein families that play a role in the tumor microenvironment. Abbreviations: CXCL1, interferon-inducible protein-1; DBF4, activator of S-phase kinase 4; GRK6, G-protein coupled receptor kinase 6; FAK, focal adhesion kinase; hsp90α, heat shock protein 90α; IGFBP5, insulin growth factor binding protein 5; IL-18, interleukin-18; IL1B, interleukin1B; MIP-1d, macrophage inflammatory protein-1d; MIP-3a, macrophage inflammatory protein-3a; MMP7, matrix metalloproteinase 7; NAP-2, neutrophil-activating peptide-2; PAK2, SPARC, secreted protein, acidic, cysteine rich; TME, tumor microenvironment.

Runt-related transcription factor 2 (RUNX2) is positively associated with tumor progression in prostate cancer [25]. LC-MS/MS analysis of secreted proteins that are upregulated after RUNX2 overexpression in prostate cancer cells revealed increased expression of a number of proteins, including basement membrane components laminins A5 and B1 [25]. A shotgun proteomic analysis of metastatic melanoma cell-line secretomes also identified the matrix proteins fibronectin and extracellular matrix protein 1, as well as the matricellular proteins SPARC (secreted protein, acidic, cysteine rich) and osteopontin [26]. Studies in which EMT was induced in Madin-Darby canine kidney cells by H-RAS demonstrated both extracellular remodeling and decreased expression of basement membrane proteins, as well as increased expression of fibronectin, biglycan and SPARC [27,28]. Lai *et al*. [29] performed an extensive analysis of changes in ECM proteins during the development of hepatocellular carcinoma. They observed upregulation of collagen type IV, VI, VII, X, XIV, XV, XVI, and XVII, of splice variants Col4a2, Col6a2, and Col6a3 and of nidogen 1, decorin, and perlecan, among other proteins, indicating the utility of ECM profiling for hepatocellular carcinoma classification and diagnosis. When compared to enchondromas, chondrosarcomas have been found to possess high levels of the carboxy-terminal pro-peptide of collagen 1A1 (PC1CP) and low levels of the carboxy-terminal pro-peptide of collagen 2A1 (PC2CP), as determined by proteomic analysis [30]. Immobilized PC2CP increased the apoptosis of primary human chondrocytes, whereas soluble PC2CP led to increased cellular migration. Furthermore, soluble PC2CP increased the expression of genes that are known to favor tumor progression, such as *VEGF* (encoding vascular endothelial growth factor) and *CXCR4* (encoding chemokine receptor CXCR4), demonstrating the critical importance of protein localization in cancer [30].

Other studies assessed ECM regulation through analysis of post-translational modifications and matrix cross-linking. Upregulation of PLOD2 (procollagen-lysine, 2-oxoglutarate 5-dioxygenase 2), a regulator of collagen stiffness, in combination with COL6A1 was found to promote bone metastasis [31]. Transglutaminase 2, a crosslinker of ECM components, was upregulated in invasive ovarian cancer cell lines [32]. The upregulation of collagen-binding proteins, notably CD44 and integrins A1, B1 and gamma3, in these invasive cell lines provided insight into how cancer cells can alter their surface receptors to adapt to the microenvironment. Matrix stiffness regulates cell behavior, and a SILAC-based study of changes in protein synthesis in cells grown on rigid or soft matrix found increased synthesis of cytoskeletal and glycolysis proteins by those cells grown on rigid surfaces [33]. Chen *et al.*[34] assessed phosphorylation of proteins during cell attachment to collagen by LC-MS/MS analysis of SILAC-labeled HeLa cells. They found that 357 proteins were differentially phosphorylated, and among these were cytoskeleton-related proteins (54 proteins), transcriptional regulators (52 proteins) and kinases or phosphatases (33 proteins). Knockdown of nine kinases and phosphatases significantly reduced cell migration. Moreover, knockdown of four of these proteins - activator of S-phase kinase (DBF4), G-protein coupled receptor kinase 6 (GRK6), p21 protein (Cdc42/Rac)-activated kinase 2 (PAK2), and focal adhesion kinase (FAK/PTK2) - also resulted in decreased invasion. Blocking of phosphorylation by mutation of the serine or threonine residues in DBF4, GRK6 or PAK2 also inhibited collagen I binding, migration and invasion, demonstrating that integrin-dependent phosphorylation regulates these proteins [34].

Proteases and their interactions with their inhibitors play a role in remodeling ECM and contribute to ECM composition. In a screen for proteins related to breast cancer metastasis to bone tissue, an inhibitor of several cathepsin proteases, cystatin-M (CST6), was observed to be downregulated [35]. Ectopic expression of CST6 suppressed metastasis in animal studies, whereas CST6 knockdown increased tumor aggressiveness [35]. Work by Blanco *et al*. [31] highlighted the interplay of ECM modulators consisting of the protease inhibitors CST1, CST2, and CST4 and the plasminogen-activating proteases PLAT and PLAU. The imbalance between matrix metalloproteinases (MMPs) and the tissue inhibitors of metalloproteinases (TIMPs) has been widely investigated [36]. Several studies have used proteomics to identify targets of membrane type 1 metalloprotease (MT1-MMP), which has been proposed to play an important role in cell invasion [37,38]. Affinity purification of MT1-MMP identified 163 proteins associated with MT1-MMP through LC-MS/MS analysis [39]. Nine membrane proteins were confirmed to be cleaved by MT1-MMP by co-expression, including the Lutheran blood group glycoprotein (Lu). Tam *et al*. [40] identified new targets of MT1-MMP through an LC-MS/MS screen of isotope-labeled proteins from a breast cancer cell line, confirming the substrates with MALDI. Interleukin-8 (IL-8) was shown to be processed by MT1-MMP to a more active form, whereas fibronectin expression was increased after MT1-MMP transfection. A novel proteomic approach, using fluorophore-assisted light inactivation (FALI) with antibody libraries for the identification of proteins related to invasiveness, revealed an extracellular form of hsp90α that acts as a chaperone for MMP2 and assists in its invasion-associated activation [41]. A comparison of high- and low-invasive ovarian cell lines confirmed that only cell lines expressing MT1-MMP were invasive through collagen-binding [41]. A targeted approach using the TRAMP (transgenic adenocarcinoma of the mouse prostate) mouse model found a general increase in the expression of ECM-modifying proteases, such as MMPs and cysteine proteases, during tumor progression [42]. Raf/MEK/ERK signaling disrupts tissue polarity in breast epithelial cells through MMP9 activity [43]. LC-MS/MS analysis of conditioned media from cells with or without active MMP9 demonstrated that the basement membrane component laminin 111 is a target of MMP9 [43]. It should be noted that proteins may be released into the microenvironment via many mechanisms. There is currently substantial interest in the role of micro-particles and exosomes, and their protein and other cargoes, as extracellular messengers whose protein content can be readily profiled by MS [14].

These studies are illustrative of the contribution of proteomic analysis to elucidating how tumor cells modify their microenvironment through the production of a diverse set of extracellular matrix proteins and proteases. A potential limitation of these studies is, however, that they do not address in a mechanistic way how the diverse mutations and genomic alterations observed in tumor cells, either singly or in combination, contribute to the capacity of these cells to alter their microenvironment. Thus, the extent to which various oncogenic drivers impact the tumor microenvironment through common or vastly distinct processes remains to be determined.

## Deciphering tumor-stroma interactions

Proteomics has provided insight into the role of CAFs and other stromal cells in tumor development (Table 1). A proteomic analysis of de-cellularized matrix from tumors with marked angiogenesis identified 50 angiogenic proteins. One such protein, galectin-1, was demonstrated to act in endothelial cell recruitment [44]. A proteomic comparison of laser capture microscopy-dissected stroma from nasopharyngeal carcinoma tumors with normal stroma identified 60 differentially expressed proteins in the tumor stroma, including the actin-regulating protein CapG, which was upregulated [45]. Reverse-phase protein microarrays targeted at signaling proteins were used to compare tumor epithelia, normal epithelia, tumor stroma and normal stroma. Interestingly, the tumor epithelia and stroma bore more similarity to each other than to normal epithelia or stroma, respectively, suggesting that EMT occurred in the epithelial tumor tissue. In particular, proteins related to the mitogen-activated protein kinase 1 (MAPK 1) pathway were more abundant in the tumor stroma and epithelia [46]. Fleming *et al.*[47] compared the mammary microenvironment of premenopausal African-American (AA) women and Caucasian-American (CAU) women, and found that the breast microenvironment in AA women was more restrictive of tumor growth. An LC-MS/MS analysis of ECM from AA and CAU women revealed differences in ECM composition (only 50% overlap of ECM proteins) and identified upregulation of actin cytoskeleton signaling in the ECM of AA women. A novel LC-MS/MS analysis also measured active hormones in the breast tissue directly, revealing higher levels of estradiol, estriol and 2-methoxyestrone in breast tissue from CAU women [47].

CAFs also contribute to the tumor microenvironment. Activated pancreatic stellate cells (PSC) promote tumor implantation, and conditioned media from PSCs enhance invasion and proliferation of cancer cells. An analysis comparing quiescent and activated PSCs found a large increase in protein secretion by the activated cells. The secreted proteins had 60% overlap with proteins from cancer cell line secretions and played a role in wound healing, inflammation, ECM and fibrosis [48]. Work by Trimmer *et al.*[49] identified loss of caveolin-1 (CAV-1) in CAFs as a predictor of clinical outcome, probably resulting from autophagy of CAFs with CAV-1. A proteomic analysis of CAV-1-deficient fibroblasts supported this hypothesis by showing upregulation of proteins related to oxidative stress. Superoxide dismutase 2 (SOD2) was identified as a suppressor of this effect by reducing oxidative stress [49].

A proteomic analysis comparing MMP7-treated and untreated colonic fibroblasts identified Insulin growth factor binding protein-5 (IGFBP-5) as a substrate of MMP7 activity [50]. MMP-7 acts as a stimulating factor in the proliferation and migration of colonic myofibroblasts through blockade of IGFBP-5 inhibition of IGF-II [50]. An MS analysis of PTEN (phosphatase and tensin homolog)-null stromal cells expressing the microRNA miR-320 revealed altered secretome expression of proteins such as MMP9, MMP2, bone morphogenic protein 1 (BMP1), lysyl oxidase-like 2 (LOXL2) and EMILIN2 when compared to a control [51]. Confirmatory studies demonstrated that blocking of MMP9 and EMILIN2 reduced the ability of conditioned media to promote the migration of epithelial cancer cells or to recruit endothelial cells, respectively. Furthermore, the secretome signature also correlated with patient outcome [51]. Newman *et al*. [52] identified factors secreted from fibroblasts that support endothelial cell lumen formation. PCOLCE, Col1A1, transforming growth factor β-inducible gene H3 (βIG-H3), SPARC, or IGFBP7 were identified in fibroblast-conditioned media fractions that were necessary for endothelial cell lumen formation [52]. Knockdown of combinations of these factors in fibroblasts inhibited endothelial cell lumen formation [52]. While stromal cells affect tumor cell behavior, there are reciprocal interactions. CAFs and tumor-adjacent fibroblasts (TAFs) in the microenvironment of breast cancer cells display features similar to those of tumor cells. CAFs and TAFs can be distinguished from normal breast fibroblasts on the basis of their proteome [53].

The co-culture of fibroblasts and cancer cells enables direct interactions to be studied. Co-culture of a murine K-ras mutant lung adenocarcinoma cell line (LKR-13) with murine lung stromal cells enhanced the migration and increased the proliferation of LKR-13 cells; co-culture also induced these cells to form epithelial tubes [54]. An LC-MS/MS analysis revealed loss of cell adhesion proteins such as E-cadherin and provided evidence of EMT in the cancer cell line when grown with fibroblasts. This LC-MS/MS analysis was supplemented by a multiplexed cytokine assay to identify signaling molecules at concentrations below the limit of detection of MS. Chemokine CXCL1 and IL-18 were found to be necessary for increased proliferation and migration [54]. Conditioned media from gastric cancer-associated myofibroblasts increased cancer cell proliferation, migration and invasion in a cancer cell line [55]. An LC-MS/MS analysis of isobaric tagged cells identified downregulation of βIG-H3 in CAFs compared to normal or adjacent myofibroblasts. βIG-H3 inhibited IGF-II-stimulated migration and proliferation of cancer cells [55].

Co-culture with stromal cells has also been shown to increase resistance to anti-cancer therapies in many cancer cell lines [56-59]. Conditioned media from some stromal lines were found to be capable of rescuing the growth of melanoma cell lines that were exposed to the Raf inhibitor vemurafenib [60]. Antibody array analysis of secreted factors, comparing media from stromal cell lines that were or were not shown to be capable of rescuing melanoma cells from vemurafenib treatment, revealed a potential role for hepatocyte growth factor (HGF), mediated through the MAPK and PI3K-AKT pathways, in resistance to vemurafenib. Head and neck squamous cell carcinoma cells and fibroblasts produce more prostaglandin PGE2 when co-cultured [61]. Moreover, co-culture of colon cancer cells with normal colon fibroblasts produced a desmoplastic set of proteins expressed solely in the co-culture but not in the individual cultures. Secreted proteins in this set included collagen type XII, a marker of the invasive front of colorectal tumors [62]. These and other studies that were based on the well-established finding that stromal cells modify the behavior of tumor cells and *vice versa* have elucidated signaling molecules, proteases and other proteins that are crucial to this interaction. The challenge is to identify the most critical factors that might be targeted by small molecule or antibody therapeutics.

## Elucidating the role of immune cells in the microenvironment through proteomics

There is intense interest in elucidating critical interactions between tumor cells and immune cells in the microenvironment. Cells with immunosuppressive potential include macrophages, regulatory T (Treg) cells and myeloid derived suppressor cells (MDSCs). Infiltrating immune cells are capable of stimulating tumor growth through the expression of signaling molecules (such as interleukins or cytokines) and growth factors (such as epidermal growth factor (EGF), TGFβ and fibroblast growth factor (FGF)), as well as through the secretion of ECM-modifying proteases [63-66]. Both antibody arrays and MS have been utilized to profile immune cells and their derived cytokines (Table 1). An antibody array was used to analyze the expression of cytokines in mesothelioma pleural effusions and in conditioned media from cell lines established from the same tumors. This study detected HGF, macrophage inflammatory protein (MIP)-1d, MIP-3a, neutrophil-activating peptide (NAP)-2, and pulmonary activation-regulated chemokine (PARC) exclusively in the pleural effusions, suggesting that these cytokines may be primarily expressed by stromal or inflammatory cells [67]. Immunohistochemistry revealed infiltration of macrophages, NK cells and T-lymphocytes in the mesothelioma tumors. Mesothelioma cell lines expressed many chemokines that seem to recruit immune cells, such as interferon-inducible protein-10 (CXCL10), macrophage migration inhibitory factor (MIF), monocyte chemoattractant protein-1 (MCP-1, also known as CCL2), epithelial neutrophil-activating protein-78 (ENA-78), MIP-1b, IL-8, growth regulatory protein (GRO) and RANTES [67]. An LC-MS/MS analysis of the cell supernatants from tumor-associated monocytes or macrophages isolated from the ascites of ovarian cancer patients identified 14-3-3 zeta, an adapter protein that potentially regulates a large number of molecules in signaling pathways [68].

Oxidative stress promotes the infiltration of inflammatory cells by providing favorable growth conditions, and these cells further contribute to the hypoxic environment by producing reactive oxygen species (ROS) [69]. A comparative proteome analysis of naive CD45RA+T cells and their memory/effector CD45RO+T cells in response to oxidative stress identified differential expression of proteins that are involved in signaling pathways, in regulating the cellular redox status and in maintaining structural cell integrity, providing a basis for therapeutic interventions to overcome oxidative stress in the tumor microenvironment [70].

MDSCs regulate immunosuppression during tumor development and cancer-associated inflammation increases the accumulation of MDSCs [71]. A proteomic analysis of MDSCs treated with IL-1B to induce inflammation identified the upregulation of the FAS pathway and caspase network [72]. Furthermore, a follow-up study revealed that inflammation reduces Fas-mediated apoptosis in MDSCs [72]. Igγ-1 chain C region (IGHG1) was uniquely identified in pancreatic cancer tissue by an LC-MS/MS analysis comparing this tissue with normal pancreatic tissue. A cell line expressing IGHG1 on its surface was generated, and when injected into a mouse pancreatic tumor model, resulted in tumors that grew more quickly than in controls, and these mice had shorter survival times. The mechanism of the increased rate of tumor growth appeared to be related to immunosuppression, as NK cells were less cytotoxic to pancreatic cancer cells that expressed IGHG1 [73]. Clearly, infiltrating immune cells may enhance tumor growth and progression through the secretion of cytokines and ECM components, and they can also help to promote a hypoxic environment.

The above studies notwithstanding, there remains a substantial need and opportunity for in-depth profiling of immune infiltrating cells and cancer cells to define their expression patterns in specific compartments (for example, in the secretome and at the cell surface) and to explore how the expression repertoire in each compartment might contribute to enhancing or suppressing tumor development and progression. Such understanding is likely to expand the repertoire of therapeutic targets for immunotherapy. Moreover, the role of post-translational modifications and their impact on the interactions between cancer cells and immune cells needs to be further explored. Besides proteomic analysis, deciphering the metabolome in the tumor microenvironment and determining how cancer cells may metabolically inhibit immune cells, allowing tumor development and cancer progression, are likely to be highly informative and complementary to proteomic profiling.

## Profiling of signaling proteins and pathways

The presence of diverse cytokines, chemokines and growth factors affects the behavior of both malignant and stromal cells. Nevertheless, elucidation of how a multitude of factors act in concert remains a challenge. Most studies have identified important roles for particular factors in a given setting. For example, conditioned media from glioblastoma multiforme (GBM) cells can transform neural precursor cells, causing them to become highly proliferative and to express markers of stem cells. LC-MS/MS analysis of GBM-conditioned media identified chitinase-3-like protein 1 (CHI3L1, also known as YKL40) and histone H2A-X, along with VEGF, EGF and platelet-derived growth factor (PDGF) [74]. The relevance of these factors in a broader setting remains to be determined. Likewise, Fukaya *et al*. [75] reported the identification of a secreted factor from neuroblastoma cells that stimulates IL-6 production in bone marrow stromal cells. Fractionation and LC-MS/MS analysis of conditioned media identified galectin-3-binding protein as the stimulating factor [75]. Conditioned media from estradiol-stimulated prostate stromal cells promote the migration of prostate cancer cells [76]. Through a MALDI-TOF analysis of conditioned media, secreted enolase 1 (ENO1) was identified and shown to be the factor responsible for increased cell migration, acting at the surface of prostate cancer cells to promote plasminogen activation [76]. An analysis of mammary fat tissue by MS and antibody arrays identified a large number of cytokines and growth factors expressed by adipocytes [77]. A broad exploration of cytokines was conducted in fluid collected from surgical wounds of breast cancer patients before and after targeted intraoperative radiotherapy treatment (TARGIT) [78]. Wound fluid from TARGIT-treated patients was less stimulatory to migration and proliferation than that from untreated patients. Cytokine analysis using an antibody array revealed reduced levels of HGF, leptin, RANTES, IL-6, IL-7, IL-8 and IL-10, while FAS, FGF-4, IL-4, IL-5, and IL-13 were upregulated [78].

## From the microenvironment to the circulation: the search for biomarkers

The microenvironment is a potentially rich source of biomarkers that are released into the circulatory system and can be mined through proteomics. Cohen *et al*. [79] performed an LC-MS/MS analysis of plasma from breast cancer patients and identified fibronectin, clusterin, gelsolin and protein AMBP (alpha-1-microglobulin/inter-α-trypsin inhibitor light chain precursor) as differentially expressed between infiltrating ductal and invasive breast cancers. Vitronectin was identified in an MS analysis comparing serum from breast ductal carcinoma *in situ* (DCIS) patients to healthy controls. Immunohistochemistry analysis of tumors and adjacent tissue revealed high expression of vitronectin in the ECM of the stroma [80]. MALDI-MS identified IGHA2 as a biomarker of microenvironment remodeling by breast tumors [81]. Elevated levels of tumor microenvironment-derived proteins in the circulation have been shown to have diagnostic potential for pancreatic cancer [82]. A proteomic analysis of plasma from mice with HER2/neu-induced mammary tumors and plasma from control mice with inflammation was performed to determine the repertoire of changes in the plasma proteome during breast tumor development. In this study, 20% of proteins that were upregulated upon tumor onset were considered likely to be associated with a tumor-specific host response, including multiple macrophage signaling proteins, TAM-related proteases, and ECM proteins [83]. Altered glycosylation of the ECM-modifying protease plasminogen was found in gastric cancer precursor lesions, suggesting its potential as a biomarker [84]. LC-MS/MS analysis of conditioned media from primary neurofibroma Schwann cells compared to Schwann cells from normal nerve tissue identified a secreted form of retinoic acid responder 1 (RARRES1) from neurofibroma Schwann cells [85].

Associations have been made between the expression of protein biomarkers identified in proteomic studies and patient prognosis. Laser-capture microdissection was used to assess proteomic changes in the stroma of nasopharyngeal carcinoma, resulting in the identification of periostin as a protein that is upregulated in these carcinoma cells when compared to normal nasopharyngeal tissue. Overexpression of periostin in nasopharyngeal carcinoma stroma was then found to be associated with advanced clinical stage [86]. In another study, expression of galectin-1 (Gal-1) in the tumor microenvironment (near non-tumor tissues) was associated with poor survival in classic Hodgkin lymphoma (cHL) patients [87].

Tissue interstitial fluid (TIF) from tumors also represents a potential source for circulating biomarkers. TIF from renal cell carcinoma and adjacent normal kidneys was compared by MS, and 138 differentially regulated proteins were identified. Enolase 2 and thrombospondin-1 were found to be upregulated in the sera of renal cell carcinoma patients [88]. Proteomic analysis of sera from mice implanted with orthotopic human oral squamous cell carcinomas allowed discrimination between host- and tumor-derived proteins. Furthermore, 31 murine proteins were identified as differentially regulated, including α-2-HS-glycoprotein, complement C3 and C4 and hemopexin [89]. Circulating proteolytic peptides cleaved by carboxypeptidase N in the tumor microenvironment have been shown to have potential for early detection of breast cancer [90]. Integrated analysis of tumor cell line secretome and plasma has provided a means for identifying potential circulating markers. A study that targeted glycoproteins secreted from the human colon carcinoma cell line LIM1215 as a source of potential colorectal carcinoma biomarkers identified a set of glycoproteins that were also found in tumor xenograft TIF and in plasma derived from mice bearing the LIM1215 xenograft tumor [91].

Although host- and microenvironment-derived proteins may well serve as cancer biomarkers, there is a considerable challenge in confirming their specificity for particular tumor types or stages of tumor development, and more broadly their cancer specificity. On the other hand, studies to date suggest that detecting signatures in plasma derived from the tumor microenvironment might provide the necessary sensitivity to determine the presence of tumors at an early stage. Thus, there is likely to be a trade-off between sensitivity and specificity in the use of microenvironment-derived markers for the early detection of tumors.

## Conclusions

The interactions between tumor cells and surrounding tissue represent a crucial area of study in the elucidation of mechanisms of cancer development, progression and metastasis, and in providing novel avenues for cancer detection and treatment. Proteomics is particularly well suited for the analysis of the microenvironment, given the host origin of numerous components of the microenvironment that lack discernible genomic alterations, and given the contribution of protein release and shedding of proteins from the surface of cancer cells that cannot be predicted strictly from genomic analysis. Proteomic analysis in particular has enhanced understanding of how tumor cells modify their microenvironment through production of ECM structural proteins, ECM-modifying proteins and proteases. Proteomics has further enhanced the global identification of protease targets. Combining MS or antibody arrays with experimental approaches, such as co-culture of cancer cells with tumor-associated fibroblasts, has also facilitated the identification of growth and signaling factors produced by stromal and infiltrating cells.

Early use of proteomics in cancer research primarily focused on the identification or quantification of proteins, and provided only modest insight into the biological relevance of the findings. As proteomic technologies become more widely utilized, targeted approaches should be applied to elucidate the function of particular proteins. Likewise, as MS technology improves through increased scanning speeds and more sensitive instruments, a larger number of identified proteins and smaller perturbations of protein abundance, along with post-translational modifications, will be observed, allowing for a more fully informative readout of protein expression. Moreover, given that a large fraction of the proteome has an enzymatic function, assessment of activity states, cleavage products and metabolic intermediates will be needed to appreciate more fully the dynamic nature of the tumor microenvironment and key targets for intervention.

## Abbreviations

CAF: Cancer-associated fibroblast; cHL: Classic hodgkin lymphoma; ECM: Extracellular matrix; EGF: Epidermal growth factor; EMT: Epithelial-mesenchymal transition; ESI: Electrospray ionization; FALI: Fluorophore-assisted light inactivation; GBM: Glioblastoma multiforme; HGF: Hepatocyte growth factor; IL: Interleukin; LC-MS/MS: Combined liquid chromatography with tandem MS; MALDI: Matrix-assisted laser desorption ionization; MDSC: Myeloid-derived suppressor cell; MMP: Matrix metalloproteinase; MS: Mass spectrometry; MS/MS: Tandem mass spectrometry; PSC: Pancreatic stellate cell; ROS: Reactive oxygen species; SILAC: Stable isotope labeling of amino acids in cell culture; TAF: Tumor-adjacent fibroblast; TARGIT: Targeted intraoperative radiotherapy treatment; TIF: Tissue interstitial fluid; TIMP: Tissue inhibitor of metalloproteinase; Treg: Regulatory T cells; VEGF: Vascular endothelial growth factor.

## Competing interests

The authors declare that they have no competing interests.
